# Cross-Cultural Adaptation and Validation of the Jebsen-Taylor Hand Function Test in an Italian Population

**DOI:** 10.1155/2016/8970917

**Published:** 2016-07-18

**Authors:** Greta Culicchia, Marta Nobilia, Marilyn Asturi, Valter Santilli, Marco Paoloni, Rita De Santis, Giovanni Galeoto

**Affiliations:** ^1^Department of Anatomical Sciences, Histological, Forensic and of the Musculoskeletal System, Sapienza University of Rome, Piazzale Aldo Moro 5, 00161 Rome, Italy; ^2^High School Science “Lazzaro Spallanzani”, Via Rivellese 1, 00019 Tivoli, Italy

## Abstract

*Objective*. This paper describes the Italian translation and adaptation to the Italian culture of the original version of the Jebsen-Taylor hand function test and conveys the procedure for testing its validity and reliability.* Design*. The cultural adaptation process and validation were based on data from a group of people with no clinical evidence of disease or impairment of the upper limbs. The process required a forward and reverse translation in its original language. The scale obtained was reviewed by 8 experts in the field of psychometrics dealing with statistical methods that are useful for the behavioral and social sciences. The Italian adapted version of the JTHFT was then produced and validated.* Participants*. The test was submitted to 320 people with no clinical evidence of disease in order to test its acceptability and consistency.* Results*. The total time required to perform each subtest was 80.16 ± 43.13 seconds for the nondominant hand (NDH) and 49.97 ± 27.28 seconds for the dominant hand (DH). The internal consistency (assessed with Pearson's *r*) and the reliability or the construct validity (assessed with Cronbach's alpha) are significative.* Conclusions*. This is the first study reporting the result of the translation, cultural adaptation, and validation protocols of the JTHFT in Italian. It provides a new tool for Italian professionals to measure the functionality of the hand in participants with various upper limb pathologies.

## 1. Introduction

Hand function evaluations are an important element for the assessment of physical rehabilitation [1]. The functional abilities depend on anatomical integrity, muscle strength, sensation, and dexterity [2]. Those elements are influenced by age, gender, and mental state [3].

The mere evaluation of these individual variables is however not suitable to assess hand function, which can only be evaluated by simulating the activities of daily living.

In clinical literature there are many tests for the hand function evaluation, such as Sollerman hand function test [4], action research arm test [5], and Toronto Rehabilitation Institute-Hand Function Test [6].

The authors chose the Jebsen-Taylor hand function test (JTHFT) [2] for the following reasons: it provides objective measurements of standardized tasks relative to norms; it evaluates broad aspects of those hand functions commonly used in everyday activities, and it can be administered in a short time by using readily available materials.

In 1969 Jebsen et al. developed the Jebsen-Taylor hand function test [2] that represents the most widely used assessment tool in rehabilitation due to its simplicity, convenience, and speed of administration.

The JTHFT is a seven-part, timed diagnostic test to evaluate the level of hand function. Each subtest was designed to test each subject in precisely the same manner. The seven subtests include writing, simulated page turning, lifting small objects, simulated feeding, stacking, and lifting large, lightweight, and heavy objects. The nondominant hand is tested before the dominant hand, and each task is timed by using a stopwatch. The measurements of the hand function are an essential element for the occupational therapist in order to outline limitations and functional capacities, to develop a proper treatment plan for each patient, to monitor the progression of the disease, and to test the effectiveness of the treatment.

We conducted a MEDLINE search for articles regarding the “Jebsen Taylor hand function test” and found more than 174 publications using this test as a mean to evaluate the outcome. Among these publications, 32 deal with cerebral stroke [7, 8], 26 with unilateral cerebral palsy [9, 10], 15 with hemiparesis [11, 12], 2 with Parkinson's disease [13, 14], 5 with tetraplegia [15, 16], 2 with spinal cord injury [17, 18], 3 with carpal tunnel syndrome [19–21], 6 with fractures [22, 23], 9 with rheumatic arthropathies [24, 25], 4 with prosthetic rehabilitation [26, 27], 3 with virtual rehabilitation [28, 29], 5 with diabetes [30, 31], 5 with burns [32, 33], 14 with manual dexterity [34, 35], 6 with splints [36, 37], and many other articles with individual diseases such as spina bifida cystica [38], Duchenne muscular dystrophy [39], and Huntington disease [40].

Indeed, although it is a text dated 1969, it is still widely used in rehabilitations, especially by those occupational therapists who assess hand dexterity while performing everyday skills. It shows good validity and reliability. Currently, within the medical literature there are several validation studies in different world languages, performed on Australian children (1982) [41], as well as Chinese (2004) [42], Portuguese (2010) [43] and American (2010) [44], and Australian (2016) [45, 46] children.

As evidenced by literature, the JTHFT being a test so widely used in clinical practice, the authors decided to conduct the study of validation in the Italian language.

In the literature, there are 5 articles regarding the methods used to validate its effectiveness in 5 different languages and at present 174 articles regarding the JTHFT protocol. The pioneering value of this study is to also validate the test in the Italian language in such a wise to be used in our country as well.

The aims of this study are the translation, cultural adaptation, and validation of the JTHFT in Italian in a group of individuals with no clinical evidence of disease in the upper limbs, in order to determine the median statistics for the Italian healthy population.

In addition, we evaluate interrater and intrarater consensus for the Italian version of the test. We furthermore analyze the relationship between the results obtained in testing for the age, gender, schooling, hobbies, and gripping force that are required before the test is administered.

## 2. Methods

The study was divided into two stages. First, the original English version of Jebsen-Taylor hand function test was translated into Italian and was culturally adapted according to a procedure met with team consensus and as described by the census bureau guideline for the translation of data collection instrument [47].

The JTHFT translated was then tested for validity and reliability in a prospective study.

### 2.1. Translation Process

The original version of the JTHFT was adopted [2]. Permission for translation, adaptation, and validation was received by the magazine that printed the original article of the test “physical medicine and rehabilitation.” The translation process included three steps. Firstly, two native English official translators have independently translated into Italian the instructions of the JTHFT (forward translation). One translator had a technical background and the other who had a medical background was responsible for evaluating the effectiveness of the translation.

Subsequently, 2 bilingual persons, blind to the original English version and independently, translated the text back into English. These two new English versions were translated into Italian by two independent health-care professionals with a certificated knowledge of the English language, blind to the original version (backward translation). All translators involved agreed to a consensus final version in order to consolidate the final translation of the JTHFT procedure.

### 2.2. Cultural Adaptation

In order to adapt the translated test to the Italian culture, it was reviewed by a panel of 8 experts in psychometric sciences, pertaining to different medical disciplines. The experts commented on the translation by writing observations on a form.

Eight experts assessed the relevance, characteristics, specificity, intelligibility, and technical quality, such as grammar or wording. The judge evaluating the translation reviewed and approved this culturally adapted final version that was then tested for soundness and reliability. The person who judged the translation reviewed and approved this final version tailored to our culture that was then tested for validity and reliability (see Appendix 1 in Supplementary Material available online at http://dx.doi.org/10.1155/2016/8970917).

### 2.3. Individual Cases and Validation Procedures

The validation process was based on data from a group of 320 healthy individuals, divided into six age groups: 6–19 (42 men and 44 women), 20–29 (39 men and 33 women), 30–39 (16 men and 17 women), 40–49 (16 men and 30 women), 50–59 (16 men and 22 women), and 60–87 (20 men and 25 women).

The subjects are free from any clinical evidence of abnormalities regarding anatomical structure, mobility, strength, sensitivity, and coordination of the upper extremities and were recruited within the national territory in the period ranging from March to September 2015. The participants of the study were all “healthy”; therefore a greater number of young people were recruited than older participants. The samples include students from elementary and secondary schools as well as students from the Sapienza University of Rome. We also recruited professional workers, housewives, and pensioners. Before administering the test, each subject was briefed and signed a written consent, releasing their personal data regarding their ages, gender, level of education, and their dominant hand.

The valid criteria for these studies were as follows: individuals between the ages of 6 and 87, recruited between March and September 2015 with a minimum level of primary education, who were able to understand instructions and perform all the tasks required from the JTHFT and who had signed the informed consent (in the case of minors, parents or legal guardians signed the consent form).

The test was administered by 2 volunteer occupational therapists (OT) and 1 physical therapist (PT). A stopwatch was used to time the completion of each test.

Before starting evaluations, all therapists were trained in the administration of the test. All statistical analyses were performed using the Statistical Package for the Social Sciences (SPSS) version 18.0 for Windows. The descriptions of the variables were carried out by using frequency tables, means, and standard deviations (SD). The data was analyzed using IBM-SPSS statistical software version 20.0.

### 2.4. Pretest (Cross-Cultural Validity)

In pretesting we wanted to determine whether any differences were present in the administration of the tests between the version in literal translation and the translation adapted culturally.

According to Perneger et al. small, approximately 5 to 15, samples taken from usual participants in pretest questionnaires may fail to uncover even the most common problems (false negatives). A default sample size of 30 participants is recommended [48]. In order to evaluate the cross-cultural validity of the JTHFT, alternatively the translated and the culturally adapted test were administered to 320 healthy individuals. To avoid bias due to translation, 50 of the 320 subjects were tested twice randomly.

The time interval between the repeated administration processes should be short enough to ensure that clinical changes have not occurred. A time interval of 6 to 7 days was considered appropriate. In pretesting we wanted to determine whether any variations were present in the administration of the tests performed from the literal translation and the tailored translation. This assessment was made using the paired sample *t*-test. The significance was found to be *p* < 0.05.

### 2.5. Reliability

*Intrarater and Interrater Reliability*. For the assessment of test-retest reliability, each patient was evaluated twice by the same professional. The time interval between test and retest should be sufficiently short in order to support the assumption that participants remain stable, yet sufficiently long to prevent recall. A time interval of 6 days was considered appropriate for the current participants.

We excluded anyone below the 6-day limit because we did not want individuals to become familiar with that particular test. In order to measure test-retest reliability, intraclass correlation coefficient (ICC) was calculated. To examine the intrarater reliability, one occupational therapist evaluated the same participant 2 times, whereas, to assess interrater reliability, two independent evaluations were made on the same person. The two operators were blind to each other during assessments. The scale was considered stable at the test-retest for ICC > 0.70. Two-way random ICC for absolute agreement was adopted to evaluate intrarater reliability [49, 50].

### 2.6. Consistency and Validity

The culturally adapted Italian JTHFT was administered to the 320 healthy individuals who signed the informed consent form [51], by the same 3 therapists who performed the cross-cultural validation and the test-retest reliability. The internal consistency was evaluated by Pearson's *r* (item for total of items). IT allowed us to evaluate Cronbach's alpha reliability and when to eliminate a given item.

The validity or the reliability methods allow us to evaluate the accuracy in determining the various subsets used to establish our protocols. We chose the measurement of the grip strength using the Jamar dynamometer. The comparison with the gold standard has been examined using Pearson's method to evaluate the correlation between the two tests.

### 2.7. Acceptability

The acceptability of the test allowed us to estimate the time of administration, the items that are not carried out, and those that were not included.

## 3. Results

### 3.1. Translation

After forward and backward translation and after a consensus meeting, the translated test was formed.

### 3.2. Cultural Adaptation

Modifications were made to individual items with the experts' opinions (see Appendix 2 in Supplementary Material).

### 3.3. Individuals

**Figure 1:**
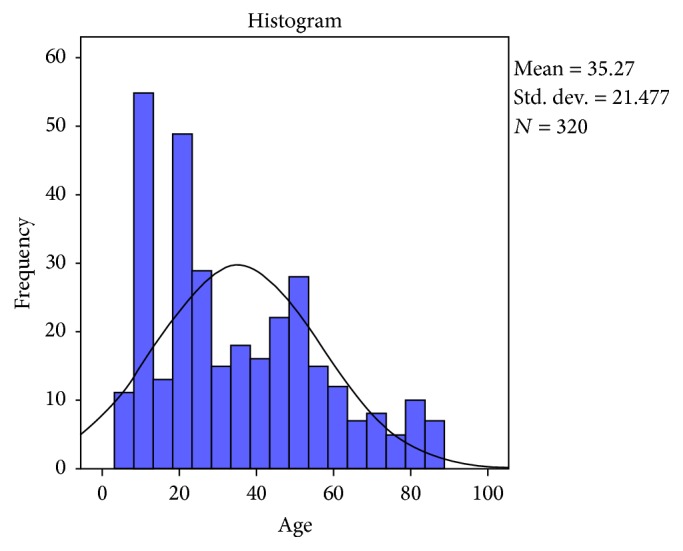
Age distribution of the participants.

The Italian culturally adapted JTHFT was administered to a total of 320 healthy individuals. Their main age was 35.27 ± 21.47 years (range 6–87) (Figure 1) of which those with a right dominant hand were 301 and those with a left dominant hand were 15. 171 subjects were females (53.4%), aged 36.58 ± 21.49 years; 149 were males (46.6%), aged 33.75 ± 21.42 years. Also, 31 (9.7%) individuals were attending primary school, 67 (20.9%) have attended primary school, 171 (53.4%) have attended secondary (higher) school, and 51 (15.9%) have graduated (Table 1).

### 3.4. Pretest (Cross-Cultural Validity)

Cross-cultural validity was evaluated on the first 60 individuals out of 320 chosen for the study (mean (DS) 48 (49.49)). The paired two-sample *t*-test revealed no significant differences between the results of the two administration processes (*t*-test = 2.603; *p* = 0.143).

### 3.5. Reliability

*Intrarater and Interrater*. 50 of the 320 included individuals were submitted to interrater and intrarater reliability procedures (female mean age 32.72 ± 3.53 and males mean age 33.14 ± 14.19). The intrarater and interrater reliability were analyzed through intraclass correlation coefficient (ICC) whose range was 0.282–0.695 for the dominant hand and 0.516–0.814 for the nondominant hand. The intrarater reliability of each subtest was analyzed by intraclass correlation coefficient (ICC), which ranged from 0.297 to 0.715 for the dominant hand and 0.584 to 0,892 for the nondominant hand (Table 2).

### 3.6. Internal Consistency and Construct Validity

The internal consistency and the construct validity were calculated on all 320 included cases. Pearson's test revealed a strong correlation between all items and between the items and the gold standard, represented by the gripping force (*p* < 0.01 and *p* < 0.05) (Tables 3 and 4).

### 3.7. Acceptability

The average time to complete each subtest was 80.16 ± 43.13 for the nondominant hand and 49.97 ± 27.28 for the dominant hand (Table 5). Two % of the sample did not bring the test completed.

### 3.8. Percentiles

Analysis of the data yielded percentiles for both males and females; level of education and age divided in two groups: 6–19 and 20–87 years. They were made to establish the normative data for the Italian population (Tables 6 [Table tab7] [Table tab8] [Table tab9] [Table tab10] [Table tab11] [Table tab12] [Table tab13] [Table tab14] [Table tab15] [Table tab16] [Table tab17] [Table tab18] [Table tab19] [Table tab20] [Table tab21] [Table tab22]–23).

## 4. Discussion

The purpose of this study was to translate the original seven items of the JTHFT into Italian, adapt it culturally to the healthy Italian population, and ultimately validate it. Translation and cultural adaptation were performed by applying internationally recognized methods [48], under the supervision of a panel of experts that ensured the maintenance of the original meaning of the items. In order to describe the differences between the translated and culturally adapted version, comparisons were made by a *t*-test analysis. The differences of total scores were not significant, indicating that the two scales could be used independently. In the first item, experts agreed that the sentences of the original tests were not appropriate for our Italian culture; therefore they have been reformulated. Also, since the Italian study introduced the age group of 6 to 19 years, issuing two new sentences of 24 letters more apt for children with a primary and secondary school education was decided, thereby facilitating comprehension, as shown in table of “adaptation of the items.” In the third test “picking up objects” two American pennies have been replaced by 2 Italian cents, both having the same diameter, so as not to maintain the same amount of grasp necessary to pick up the item. In item four “simulate power” only those beans were used that are available in Italy. The ICCs of the interrater and intrarater reliabilities of the JTHFT were satisfactory (Table 2).

Among the seven subtests, the one regarding the writing for the dominant hand had relatively high interrater and intrarater reliabilities (Table 2).

It may be due to the fact that the original instructions and procedures did not prescribe any particular font, thereby allowing the subjects to choose between cursive and print.

As a matter of fact, individuals tend to write in capital letters with their nondominant hand and in cursive mode with the dominant hand, thus obtaining different results at different times. Moreover, the instructions do not require that the pen be held in any given way.

The writing speed may also be influenced by the education level of each subject. In fact, it was observed that individuals with a lower education level had the tendency to look several times at the phrase as they wrote it, increasing the required time in which to accomplish the task.

Even the items regarding “simulated feeding” achieved a reliable correlation of test-retest and interrater relatively lower than the nondominant hand. It may be due to the lack of enforcement of any particular way in which to hold the spoon.

Internal consistency was measured by Pearson's correlation, which was statistically significant with *p* < 0.01 for the nondominant hand and *p* < 0.01 and *p* < 0.05 for the dominant hand (Table 3). Even the validity of the test was obtained through Pearson's correlation being statistically significant with *p* < 0.01 for the nondominant hand and with *p* < 0.01 and *p* < 0.05 for the dominant hand (Table 4). Among the seven items, only the third task, “picking up small common objects,” is not statistically significant for either hand. This might be related to the fact that the grasp's strength is not relevant for those activities that require grasping and handling of small objects, whereas the control of the movements is the major factor involved in the accomplishment of tasks that require precise movements.

## 5. Conclusions

Hand function is of great significance in many activities of daily living that require good coordination. The seven items of the JTHFT have been designed with the aim of providing quantitative measurements able to assess broad aspects of hand function employed in everyday life. The use of standardized assessment instruments offers many advantages to both doctors and rehabilitation professionals. These, in fact, can facilitate medical diagnosis, determine the stages of development and functional levels, plan interventions, and evaluate the effectiveness of treatment. The JTHFT is the most common assessment instrument used in rehabilitation, especially by occupational therapists, thanks to its simplicity, practicality, and speed of administration. It is widely used in rehabilitation in many countries of the world; in fact, validation studies have been carried out in different languages such as Australian, Chinese, and Portuguese in addition to the English language originally used. The JTHFT culturally adapted to the Italian language has proven to be a reliable and valid assessment instrument to measure the hand function through common activities of daily life. Also, the addition of the grip-strength measurement taken by Jamar dynamometer has shown that there is a strong correlation between the hand function and its strength. A low gripping force contributes to a decreased functional capability. On the contrary, the measure alone of applied force is not paramount to the hand functionality.

One of the limitations of the JTHFT could be that the test assesses the speed of execution of each subtest without evaluating the different strategies implemented by each individual. Developing alternative strategies allows a patient with upper limb disorders to achieve a certain degree of autonomy and independence in all areas of life. The main advantage is that the JTHFT is capable of providing an objective measure of the functionality of the hand, using standardized tasks commonly used in everyday activities. Also, the JTHFT is a test readily available, the materials are not expensive, and the time of its administration is short, and hence it is feasible in daily practice. We therefore decided to develop the manual of the JTHFT in the Italian version in order to obtain higher interrater and intrarater reliability. The procedures, the verbal instructions, and the materials for the proper administration of the JTHFT have all been reported in the manual.

*Limits of the Study*. It was not possible to establish a stratification for two age groups because the samples were not representative.

## Supplementary Material

The additional material includes the manual for administering the test.

## Figures and Tables

**Table 1:** Demographic characteristics of individuals submitted to each test.

	Pretest (*n* = 60)	Test-retest (*n* = 50)	Internal consistency (*n* = 320)
Demographic characteristics			
Age (mean ± SD)	48 ± 49.49	43 ± 24.04	35.27 ± 21.47
Female *n* (%)	28 (47%)	25 (50%)	171 (53.4%)
Educational instruction *n* (%)			
Attending elementary school	4 (7%)	0	31 (9.70%)
Elementary school	13 (22%)	2 (4%)	67 (20.90%)
Secondary school	37 (61%)	36 (72%)	171 (53.4%)
Degree	6 (10%)	12 (24%)	51 (15.90%)

**Table 2:** Interrater/intrarater reliability for dominant hand and nondominant hand.

	Interrater				Intrarater			
	DH	IC 95%	NDH	IC 95%	DH	IC 95%	NDH	IC 95%
1	0.437	(0.183–0.635)	0.673	(0.487–0.800)	0.545	(0.227–1.681)	0.787	(0.509–2.352)
2	0.824	(0.709–0.896)	0.665	(0.476–0.795)	0.930	(0.729–2.727)	0.766	(0.446–0.769)
3	0.533	(0.301–0.705)	0.547	(0.319–0.715)	0.614	(0.315–0.719)	0.643	(0.325–0.721)
4	0.561	(0.337–0.725)	0.251	(−0.027–0.493)	0.687	(0.352–0.740)	0.343	(−0.030–0.543)
5	0.782	(0.646–0.870)	0.750	(0.598–0.850)	0.865	(0.667–0.891)	0.892	(0.638–0.865)
6	0.888	(0.811–0.935)	0.901	(0.831–0.942)	0.995	(0.823–0.947)	0.995	(0.847–0.965)
7	0.824	(0.709–0.896)	0.712	(0.542–0.826)	0.892	(0.700–0.890)	0.804	(0.547–0.854)

Total	0.518	(0.282–0.695)	0.693	(0.516–0.814)	0.632	(0.297–0.715)	0.803	(0.584–0.892)

**Table 3:** Pearson's correlation for internal consistency.

	Nondominant hand							Dominant hand						
	Item 1	Item 2	Item 3	Item 4	Item 5	Item 6	Item 7	Item 1	Item 2	Item 3	Item 4	Item 5	Item 6	Item 7
Item 1	1	.439^*∗∗*^	.391^*∗∗*^	.257^*∗∗*^	.389^*∗∗*^	.275^*∗∗*^	.404^*∗∗*^	1	.367^*∗∗*^	.356^*∗∗*^	.140^*∗*^	.493^*∗∗*^	0.306	.339^*∗∗*^
Item 2	.439^*∗∗*^	1	.553^*∗∗*^	.145^*∗∗*^	.529^*∗∗*^	.610^*∗∗*^	.606^*∗∗*^	.367^*∗∗*^	1	.570^*∗∗*^	.273^*∗∗*^	.669^*∗∗*^	.664^*∗∗*^	0.684
Item 3	.391^*∗∗*^	.553^*∗∗*^	1	.231^*∗∗*^	.437^*∗∗*^	.505^*∗∗*^	.577^*∗∗*^	.356^*∗∗*^	.570^*∗∗*^	1	.319^*∗∗*^	.526^*∗∗*^	.591^*∗∗*^	.570^*∗∗*^
Item 4	.257^*∗∗*^	.145^*∗∗*^	.231^*∗∗*^	1	0.066	.180^*∗∗*^	.212^*∗∗*^	.140^*∗*^	.273^*∗∗*^	.319^*∗∗*^	1	.236^*∗∗*^	.238^*∗*^	.292^*∗∗*^
Item 5	.389^*∗∗*^	.529^*∗∗*^	.437^*∗∗*^	0.066	1	.524^*∗∗*^	.485^*∗∗*^	.493^*∗∗*^	.669^*∗∗*^	.526^*∗∗*^	.236^*∗∗*^	1	.587^*∗∗*^	.577^*∗∗*^
Item 6	.275^*∗∗*^	.610^*∗∗*^	.505^*∗∗*^	.180^*∗∗*^	.524^*∗∗*^	1	.817^*∗∗*^	.306^*∗∗*^	.664^*∗∗*^	.591^*∗∗*^	.238^*∗∗*^	.587^*∗∗*^	1	.854^*∗∗*^
Item 7	.404^*∗∗*^	.606^*∗∗*^	.577^*∗∗*^	.212^*∗∗*^	.485^*∗∗*^	.817^*∗∗*^	1	.339^*∗∗*^	.684^*∗∗*^	.570^*∗∗*^	.292^*∗∗*^	.577^*∗∗*^	.854^*∗∗*^	1

^*∗∗*^Correlation is significant at the 0.01 level (2-tailed).

^*∗*^Correlation is significant at the 0.05 level (2-tailed).

**Table 4:** Pearson's correlation for construct validity.

Jamar dynamometer		
	NDH	DH
Item 1	−.142^*∗∗*^	−.176^*∗∗*^
Item 2	−.172^*∗∗*^	−.167^*∗∗*^
Item 3	−0.241	−0.209
Item 4	−.378^*∗∗*^	−.364^*∗∗*^
Item 5	−.053^*∗∗*^	−.145^*∗∗*^
Item 6	−.161^*∗∗*^	−.178^*∗∗*^
Item 7	−.262^*∗∗*^	−.243^*∗∗*^

^*∗∗*^Correlation is significant at the 0.01 level (2-tailed).

**Table 5:** Mean of time and SD.

Nondominant hand	Mean	St. deviation	Dominant hand	Mean	St. deviation
Item 1	43.7	31.4	Item 1	18.8	18.6
Item 2	5.0	1.6	Item 2	4.5	1.5
Item 3	6.8	1.4	Item 3	6.3	1.3
Item 4	11.6	5.4	Item 4	8.8	2.8
Item 5	4.7	1.3	Item 5	4.1	1.1
Item 6	4.1	1.1	Item 6	3.7	0.9
Item 7	4.1	1.1	Item 7	3.8	0.9

Total	80.2	43.1	Total	49.9	27.3

**Table 6:** *n* = 15, males, 6–19, nondominant hand, educational instruction: attending elementary school.

Percentiles	Item 1	Item 2	Item 3	Item 4	Item 5	Item 6	Item 7	Dynamometer
90	22.586	3.366	4.776	10.34	2.988	3.298	2.988	36.92
80	28.526	3.91	6.212	11.746	3.16	3.47	3.368	31.54
70	33.454	4.238	7.332	12.474	3.712	3.59	3.888	29.38
60	36.978	4.476	7.59	13.834	4.268	3.726	4.118	25.72
50	39.53	4.62	7.81	15.06	4.47	3.88	4.16	24.3
40	44.138	5.05	7.984	17.768	4.726	4.096	4.376	23.28
30	47.364	6.4	8.31	19.244	4.916	4.256	4.488	20.7
20	55.212	7.024	8.814	24.172	5.068	4.408	4.792	17.26
10	113.654	7.908	9.336	45.562	5.374	4.69	5.248	15.6

**Table 7 tab7:** *n* = 16, females, 6–19, nondominant hand, educational instruction: attending elementary school.

Percentiles	Item 1	Item 2	Item 3	Item 4	Item 5	Item 6	Item 7	Dynamometer
90	18.349	3.579	5.539	9.957	3.353	3.232	3.476	24.5
80	24.132	3.904	5.952	10.924	3.492	3.446	3.594	21.7
70	37.068	3.94	6.239	12.128	3.793	3.66	3.615	21.27
60	40.732	4.412	6.634	13.68	3.982	3.75	3.822	20.76
50	41.75	4.74	6.985	15.92	4.06	4	4.185	20.5
40	42.714	5.068	7.264	17.882	4.094	4.426	4.36	19.8
30	43.948	5.364	7.719	20.908	4.388	4.659	4.494	19
20	52.942	5.872	8.9	21.842	5.03	5.43	4.926	17.68
10	65.41	6.472	9.916	25.145	5.226	5.954	5.702	13.6

**Table 8 tab8:** *n* = 21, females, 6–19, nondominant hand, educational instruction: elementary school.

Percentiles	Item 1	Item 2	Item 3	Item 4	Item 5	Item 6	Item 7	Dynamometer
90	25.884	3.838	5.252	9.154	3.164	3.392	3.58	34.24
80	28.692	4.244	6.208	10.166	3.576	3.614	3.968	32.9
70	30.52	4.37	6.494	10.578	4.096	3.894	4.238	31.94
60	32.114	4.504	6.762	11.338	4.424	4.116	4.376	29.5
50	32.6	4.62	7.12	11.72	4.68	4.22	4.57	29
40	35.12	4.75	7.62	13.036	4.788	4.518	4.87	25.24
30	36.966	5.32	7.964	14.274	4.874	4.726	5.072	24.18
20	39.098	5.848	8.166	15.03	5.006	5.228	5.388	22.86
10	49.862	7.5	8.736	15.888	6.662	5.87	6.338	21.2

**Table 9 tab9:** *n* = 19; males, 6–19, nondominant hand, educational instruction: elementary school.

Percentiles	Item 1	Item 2	Item 3	Item 4	Item 5	Item 6	Item 7	Dynamometer
90	22.78	3.38	5.34	7.59	3.13	2.94	3.09	71
80	27.97	3.56	5.43	10.31	3.28	3.06	3.28	59.3
70	31.46	3.91	6.22	10.84	3.56	3.25	3.43	58.3
60	33	3.94	6.47	11.75	3.69	3.28	3.5	56
50	34.25	3.97	6.78	13.85	3.84	3.35	3.65	54.7
40	37.94	4.03	7.13	14.91	4	3.54	3.66	49.7
30	40.72	4.25	7.56	17.4	4.31	3.69	3.81	47
20	42.66	4.56	7.82	20.38	4.65	3.82	4	42
10	48.47	5.28	7.91	25.34	5.25	3.94	4.22	39.7

**Table 10 tab10:** *n* = 19, females, 20–94, nondominant hand, educational instruction: elementary school.

Percentiles	Item 1	Item 2	Item 3	Item 4	Item 5	Item 6	Item 7	Dynamometer
90	50.59	4.41	6.15	8.93	4.25	3.56	3.72	55.7
80	72.07	4.96	6.97	10.94	4.5	3.69	3.84	46.3
70	79.19	5.72	7.18	11.56	5	4.13	4.16	45
60	89.2	5.84	7.69	12.03	5.31	4.37	4.41	41.3
50	102.74	5.93	8.43	12.31	5.5	4.72	4.65	37
40	124.53	6.85	8.94	13.41	5.72	4.78	4.91	33
30	136.13	7.47	9.59	13.65	6.75	5.03	5.09	30.3
20	158.78	7.68	9.71	15.88	7.1	5.06	6.09	26.7
10	174.57	9.84	10.19	17	7.41	5.87	7.82	23.3

**Table 11 tab11:** *n* = 74, females, 20–94, nondominant hand, educational instruction: secondary school.

Percentiles	Item 1	Item 2	Item 3	Item 4	Item 5	Item 6	Item 7	Dynamometer
90	22.52	3.545	5.265	8.39	3.59	3.08	3.11	70.8
80	24.34	3.88	5.69	8.97	3.72	3.22	3.28	66
70	27.19	4.09	5.895	9.25	3.97	3.405	3.42	59.85
60	29.78	4.28	6.12	9.5	4.13	3.5	3.59	56.7
50	31.525	4.53	6.28	10.095	4.405	3.61	3.78	52.7
40	34.28	4.69	6.53	10.6	4.69	3.81	3.91	49.7
30	38.405	4.915	6.795	10.93	5.065	4.075	4.105	42.65
20	40.87	5.35	7.13	11.88	5.32	4.62	4.53	38.3
10	47.135	6.31	7.58	13.655	6.265	4.925	5.105	32.85

**Table 12 tab12:** *n* = 82, males, 20–94, nondominant hand, educational instruction: secondary school.

Percentiles	Item 1	Item 2	Item 3	Item 4	Item 5	Item 6	Item 7	Dynamometer
90	22.443	3.708	5.136	7.117	3.509	3.009	2.963	130.51
80	28.236	3.896	5.446	7.786	3.846	3.168	3.186	110.4
70	30.975	4.07	5.777	8.145	3.969	3.398	3.338	100.37
60	33.74	4.346	6.036	8.708	4.06	3.59	3.5	97.7
50	36.96	4.575	6.28	9.13	4.295	3.69	3.6	95.15
40	41.976	4.828	6.738	9.586	4.926	3.872	3.744	91.38
30	46.466	5.036	7.049	10.816	5.43	4.09	3.94	81.66
20	51.862	5.63	7.38	11.826	5.732	4.41	4.28	72.62
10	62.197	7.199	8.364	13.624	6.374	5.376	5.167	68.3

**Table 13 tab13:** *n* = 34, females, 20–94, nondominant hand, educational instruction: degree.

Percentiles	Item 1	Item 2	Item 3	Item 4	Item 5	Item 6	Item 7	Dynamometer
90	24.375	3.73	4.915	7.655	3.83	3.135	3.215	66.8
80	26.09	4.07	5.84	8.06	4.09	3.22	3.37	62
70	27.735	4.55	6.015	8.605	4.5	3.48	3.595	56.85
60	30.06	4.75	6.18	8.91	4.59	3.72	3.84	55
50	32.375	4.815	6.33	9.355	4.895	4.11	4.065	51.15
40	34.57	5.28	6.53	9.65	5.1	4.22	4.37	46.7
30	37.91	5.56	7.045	10.895	5.42	4.625	4.655	44.5
20	39.31	5.78	7.5	11.85	5.81	4.87	4.94	42.7
10	45.605	6.86	7.94	13.545	6.58	5.415	5.095	41.15

**Table 14 tab14:** *n* = 17, males, 20–94, nondominant hand, educational instruction: degree.

Percentiles	Item 1	Item 2	Item 3	Item 4	Item 5	Item 6	Item 7	Dynamometer
90	20.514	3.054	5.116	7.764	2.934	3.006	2.828	121.98
80	26.486	3.34	5.316	8.24	3.346	3.368	3.144	111.08
70	27.842	3.804	5.892	8.446	3.684	3.516	3.386	107.38
60	31.14	3.974	6.066	8.812	4.06	3.572	3.47	102.36
50	34.03	4.07	6.13	9.06	4.21	3.84	3.7	97
40	34.994	4.114	6.184	9.348	4.324	4.088	3.926	93.6
30	35.526	4.214	6.482	10.022	4.64	4.286	4.168	87.42
20	38.356	5.67	7.694	13.338	5.398	5.734	5.524	76.3
10	56.404	7.404	12.646	13.654	6.44	7.826	7.09	61.5

**Table 15 tab15:** *n* = 15, males, 6–19, dominant hand, educational instruction: attending elementary school.

Percentiles	Item 1	Item 2	Item 3	Item 4	Item 5	Item 6	Item 7	Dynamometer
90	10.866	3.018	5.222	9.062	2.996	2.244	2.68	48.06
80	11.502	3.324	5.878	9.494	3.178	2.964	3.196	43.3
70	11.934	3.524	6.302	10.566	3.25	3.34	3.348	40.58
60	13.4	4.14	6.534	11.572	3.606	3.576	3.57	33.1
50	14.03	4.68	6.75	12.32	4.09	3.65	3.88	29
40	17.274	5.15	6.94	12.882	4.25	3.716	3.946	28.46
30	19.564	5.5	7.53	15.274	4.354	4.042	4.038	24.54
20	25.244	5.572	7.986	19.554	4.906	4.146	4.286	22.38
10	40.648	6.356	8.46	20.638	5.336	5.024	4.734	21.42

**Table 16 tab16:** *n* = 16, females, 6–19, dominant hand, educational instruction: attending elementary school.

Percentiles	Item 1	Item 2	Item 3	Item 4	Item 5	Item 6	Item 7	Dynamometer
90	11.734	3.491	5.672	7.19	2.943	3.096	3.211	33.81
80	13.482	3.602	5.822	7.836	3.19	3.268	3.262	32.18
70	13.562	3.623	6.135	9.444	3.31	3.422	3.485	31.63
60	14.296	3.682	6.92	10.022	3.772	3.586	3.756	30.76
50	15.395	3.935	7.355	10.265	3.885	3.86	4.075	29.35
40	16.366	4.232	7.52	11.276	3.958	3.97	4.226	28.3
30	17.815	4.622	7.681	12.916	4.255	4.024	4.313	27.76
20	18.554	5.084	8.59	13.596	4.806	4.222	4.508	25.34
10	22.233	5.307	9.181	15.143	5.126	4.605	4.927	17.49

**Table 17 tab17:** *n* = 21, males, 6–19, dominant hand, educational instruction: elementary school.

Percentiles	Item 1	Item 2	Item 3	Item 4	Item 5	Item 6	Item 7	Dynamometer
90	10.4	3.372	5.312	6.692	2.684	3.048	3.33	41.62
80	11.036	3.53	5.546	7.432	3.334	3.166	3.528	40.6
70	11.376	3.666	5.792	7.966	3.696	3.548	3.852	39.12
60	11.878	4.08	5.976	8.586	3.81	3.754	4.072	38.38
50	13.31	4.47	6.22	8.72	3.97	3.85	4.19	37
40	14.142	4.87	6.398	9.02	4.2	3.978	4.42	33.94
30	14.896	5.178	6.594	10.244	4.392	4.596	4.65	31.6
20	16.442	5.932	6.816	11.628	4.782	4.864	5.168	30.58
10	17.344	6.924	7.4	14.318	5.304	5.018	5.706	28.76

**Table 18 tab18:** *n* = 19, males, 6–19, dominant hand, educational instruction: elementary school.

Percentiles	Item 1	Item 2	Item 3	Item 4	Item 5	Item 6	Item 7	Dynamometer
90	10.31	3	4.94	6.47	2.41	2.91	2.9	68.7
80	11.15	3.13	5	8.4	2.93	2.97	3.06	66.7
70	12	3.28	5.35	8.53	3.09	3.09	3.15	64.3
60	12.5	3.44	5.94	9.22	3.25	3.12	3.25	62.7
50	13.53	3.62	6.25	10.69	3.28	3.19	3.28	60.3
40	14.03	3.79	6.47	11.56	3.56	3.22	3.35	57
30	15	4.12	6.62	12	3.71	3.34	3.41	55.7
20	15.88	4.5	7.28	15.38	3.84	3.41	3.5	50
10	17.37	4.84	7.65	17.69	4.75	4.12	3.75	48.7

**Table 19 tab19:** *n* = 19, females, 20–94, dominant hand, educational instruction: elementary school.

Percentiles	Item 1	Item 2	Item 3	Item 4	Item 5	Item 6	Item 7	Dynamometer
90	29.15	4.03	5.57	6.06	3.31	3.31	3.16	60.7
80	31.31	4.31	6.29	7.19	4	3.66	3.53	56.3
70	37.88	5.07	6.53	7.68	4.53	3.79	3.97	45
60	47.44	5.31	6.94	8.22	4.87	4.03	4.31	42.3
50	52.03	5.85	7.41	8.28	5.35	4.25	4.44	39.7
40	83.75	6.07	7.66	8.68	5.59	4.75	4.77	36.3
30	97.44	6.53	8	8.87	6.18	4.97	4.84	33.7
20	105.09	7.63	8.66	9.97	6.34	5.13	4.85	30.7
10	115.84	8.03	9.18	13.6	7.28	5.53	6.4	26

**Table 20 tab20:** *n* = 74, females, 20–94, dominant hand, educational instruction: secondary school.

Percentiles	Item 1	Item 2	Item 3	Item 4	Item 5	Item 6	Item 7	Dynamometer
90	9.545	3.25	4.765	6.11	2.985	2.765	2.94	78.35
80	10.41	3.41	5.28	6.44	3.31	2.97	3.07	71.3
70	10.83	3.56	5.47	6.95	3.44	3.1	3.15	65.2
60	11.31	3.75	5.63	7.22	3.59	3.25	3.31	58.7
50	11.75	3.97	5.78	7.41	3.735	3.405	3.515	57.7
40	12.88	4.28	6.07	7.83	3.94	3.53	3.6	56
30	13.53	4.565	6.295	8.37	4.16	3.705	3.81	50.5
20	15.09	4.81	6.53	8.85	4.47	4.12	4.22	44
10	17.185	5.8	6.995	9.655	5.025	4.385	4.585	36.5

**Table 21 tab21:** *n* = 82, 20–94, dominant hand, educational instruction: secondary school.

Percentiles	Item 1	Item 2	Item 3	Item 4	Item 5	Item 6	Item 7	Dynamometer
90	8.955	3.169	4.837	6.148	2.942	2.789	2.873	74.9
80	10.858	3.362	5.262	6.488	3.144	2.996	3	83.06
70	11.461	3.623	5.5	6.895	3.38	3.12	3.099	90.27
60	13.316	3.756	5.622	7.282	3.612	3.25	3.22	95.7
50	14.3	3.92	5.84	7.47	3.84	3.345	3.36	101.35
40	15.19	4.226	6.104	7.89	4.078	3.494	3.47	113.3
30	17.487	4.609	6.506	8.313	4.287	3.684	3.693	120.7
20	19.406	5.15	6.826	8.862	5.012	3.928	3.888	124
10	23.425	5.804	7.376	10.483	5.603	4.918	4.364	128.49

**Table 22 tab22:** *n* = 34, females, 20–94, dominant hand, educational instruction: degree.

Percentiles	Item 1	Item 2	Item 3	Item 4	Item 5	Item 6	Item 7	Dynamometer
90	8.66	3.64	4.7	5.955	3.28	2.875	3.085	70.35
80	9.4	3.91	5.37	6.12	3.47	3.19	3.18	68.7
70	10.03	4.09	5.5	6.52	3.73	3.345	3.315	62.7
60	11.03	4.22	5.72	7.31	3.96	3.62	3.56	60.7
50	11.87	4.39	6.03	7.515	4.015	3.765	3.78	58.35
40	12.03	4.5	6.19	8.06	4.28	3.82	3.85	56.3
30	12.84	4.81	6.375	8.42	4.405	4.015	4.125	54.85
20	13.37	5.15	6.43	9.41	4.63	4.47	4.25	52
10	14.66	5.735	6.64	10.66	4.765	4.725	4.795	43

**Table 23:** *n* = 17, males, 20–94, dominant hand, educational instruction: degree.

Percentiles	Item 1	Item 2	Item 3	Item 4	Item 5	Item 6	Item 7	Dynamometer
90	8.772	2.628	4.748	5.712	2.638	2.554	2.506	128.9
80	9.548	2.894	4.912	6.092	2.846	2.976	3.042	121.1
70	9.998	3.03	5.064	6.522	3.076	3.096	3.226	119.54
60	10.854	3.178	5.306	6.944	3.164	3.238	3.28	112.42
50	11.56	3.44	5.75	7.12	3.44	3.47	3.28	103
40	12.394	3.78	6.844	7.736	3.66	3.664	3.602	98.84
30	13.184	4.08	7.048	7.99	4.018	4.048	3.954	89.7
20	14.414	4.642	7.734	9.548	4.32	4.696	4.636	86.72
10	17.958	8.402	10.23	12.15	5.688	7.068	6.912	81.64

## Abbreviations

JTHFT: Jebsen-Taylor hand function test
DH: Dominant hand
NDH: Nondominant hand
Adl: Activities of daily living
SPSS: Statistical Package for the Social Sciences
OT: Occupational therapist
PT: Physical therapist
SD: Standard deviations
ICC: Interclass correlation coefficient.
