# Supplementary material for: Cross-Cultural Adaptation and Validation of the Jebsen-Taylor Hand Function Test in an Italian Population
Source: Rehabil Res Pract. 2016 Jul 18;2016:8970917. doi: 10.1155/2016/8970917 (PMC4967698; doi:10.1155/2016/8970917)
Supplement: Supplementary file 1 — The additional material includes the manual for administering the test. [file 8970917.f1.docx]

**Appendix 1**

Dear therapist,

in order to arrive at a final version of the Jebsen-Taylor Hand Function Test as much as possible clear and easy to complete for authors, please for each item, to report and comment on any unclear points (of doubtful or ambiguous interpretation) difficulties that may arise during compilation (eg. the difficulty in framing a patient's condition between the options), sentences that may need clarification or for any modifications, or other comments or suggestions you deem necessary.

**Item 1:Writing**

Observations: ___________________________________________________________________________­

**Item 2: Simulated page turning**

Observations: ___________________________________________________________________________­

**Item 3: Lifting small objects**

Observations: ___________________________________________________________________________

**Item 4: Simulated feeding**

Observations: ___________________________________________________________________________

**Item 5: Stacking**

Observations: ___________________________________________________________________________

**Item 6: Lifting large, lightweight objects**

Observations ___________________________________________________________________________

**Item 7: Lifting large, heavy objects**

Observations: ___________________________________________________________________________

**Appendix 2**

| **Translated and adapted version of Italian JTHFT at comparison** | |
| --- | --- |
| **TRANSLETED ITEMS** | **ADAPTED ITEMS** |
| **ITEM 1 "** **writing "** | |
| "John vide arrivare il camion rosso" | "Mario vide arrivare il camion" |
| "Il vecchio sembrava stanco" | "Il signore sembrava nervoso" |
|  | "La mamma mi prepara una torta" |
|  | "Il cavallo mangia una carota" |
| **ITEM 2 "** **simulated page turning "** | |
| no difference | no difference |
| **ITEM 3 "** **lifting small objects "** | |
| 2 pounds americani | 2 centesimi italiani (europei) |
| **ITEM 4 "** **simulated feeding "** | |
| fagioli americani | fagioli italiani |
| **ITEM 5 "** **stacking "** | |
| no difference | no difference |
| **ITEM 6 "** **lifting large and lightweight objects** **"** | |
| lattine vuote standard n° 303 | lattine vuote 62 g, circonferenza 22 cm, diametro 7,5 cm, altezza 10,5 cm |
| **ITEM 7 "** **lifting large and heavy objects "** | |
| lattine piene standard n° 303 | lattine piene 463 g, circonferenza 22 cm, diametro 7,5 cm, altezza 10,5 cm |
